# Supplementary material for: Excitatory neurotransmission activates compartmentalized calcium transients in Müller glia without affecting lateral process motility
Source: eLife. 2021 Dec 16;10:e73202. doi: 10.7554/eLife.73202 (PMC8806189; doi:10.7554/eLife.73202)
Supplement: Transparent reporting form [file elife-73202-transrepform1.docx]

***eLife’s* transparent reporting form**

We encourage authors to provide detailed information *within their submission* to facilitate the interpretation and replication of experiments. Authors can upload supporting documentation to indicate the use of appropriate reporting guidelines for health-related research (see [EQUATOR Network](http://www.equator-network.org/%20)), life science research (see the [BioSharing Information Resource](https://biosharing.org/)), or the [ARRIVE guidelines](http://www.plosbiology.org/article/info:doi/10.1371/journal.pbio.1000412) for reporting work involving animal research. Where applicable, authors should refer to any relevant reporting standards documents in this form.

If you have any questions, please consult our Journal Policies and/or contact us: [editorial@elifesciences.org](mailto:editorial@elifesciences.org).

**Sample-size estimation**

- You should state whether an appropriate sample size was computed when the study was being designed
- You should state the statistical method of sample size computation and any required assumptions
- If no explicit power analysis was used, you should describe how you decided what sample (replicate) size (number) to use

Please outline where this information can be found within the submission (e.g., sections or figure legends), or explain why this information doesn’t apply to your submission:

No statistical power analysis was used to predetermine sample size. We determined final sample size based on pilot data, previous experience, and according to accepted practice in the field. Sample sizes are listed in figure supplements.

**Replicates**

- You should report how often each experiment was performed
- You should include a definition of biological versus technical replication
- The data obtained should be provided and sufficient information should be provided to indicate the number of independent biological and/or technical replicates
- If you encountered any outliers, you should describe how these were handled
- Criteria for exclusion/inclusion of data should be clearly stated
- High-throughput sequence data should be uploaded before submission, with a private link for reviewers provided (these are available from both GEO and ArrayExpress)

Please outline where this information can be found within the submission (e.g., sections or figure legends), or explain why this information doesn’t apply to your submission:

Each experiment was repeated in multiple independent samples/animals, with multiple cells or fields of view from each animal. For calcium imaging experiments, investigators were not blinded as results were quantitative and did not require subjective interpretation. For experiments in which glial morphology was quantified, the experimenter was blinded to experimental condition and genotype during image analysis but not during image acquisition. Whenever possible, data was processed and analyzed using automated pipelines applied to all conditions and replicates. Outliers were not excluded. Calcium imaging data was excluded if retinal waves did not occur during the acquisition, thereby preventing visualization of wave-induced responses in glia.

**Statistical reporting**

- Statistical analysis methods should be described and justified
- Raw data should be presented in figures whenever informative to do so (typically when N per group is less than 10)
- For each experiment, you should identify the statistical tests used, exact values of N, definitions of center, methods of multiple test correction, and dispersion and precision measures (e.g., mean, median, SD, SEM, confidence intervals; and, for the major substantive results, a measure of effect size (e.g., Pearson's r, Cohen's d)
- Report exact p-values wherever possible alongside the summary statistics and 95% confidence intervals. These should be reported for all key questions and not only when the p-value is less than 0.05.

Please outline where this information can be found within the submission (e.g., sections or figure legends), or explain why this information doesn’t apply to your submission:

Statistical methods are in ‘Materials and methods’ subsection ‘Statistical analysis’ as well as in figure supplements and their legends. Raw data are shown in figures wherever possible as individual dots. Results are presented as mean ± SEM, or as median [1^st^ quartile, 3^rd^ quartile] for nonparametric comparisons, as specified in ‘Materials and methods’ and in statistical tables. In most cases, summary data are plotted as box plots denoting median, 25%-75% interquartile range, and non-outlier maximum and minimum. Nonparametric statistical tests were used for glial process count data to assess changes in motility and to assess changes in intercompartment calcium transient latency. Parametric tests were used to assess glial participation in retinal waves, calcium transient amplitudes, retinal wave properties, and glial morphological measurements in wild type vs. β2-nAChR-knockout. Tests and their corresponding p-values and N’s are specified in statistical tables at the end of the text. Multiple testing correction was performed using Benjamini-Hochberg correction when necessary.

(For large datasets, or papers with a very large number of statistical tests, you may upload a single table file with tests, Ns, etc., with reference to sections in the manuscript.)

**Group allocation**

- Indicate how samples were allocated into experimental groups (in the case of clinical studies, please specify allocation to treatment method); if randomization was used, please also state if restricted randomization was applied
- Indicate if masking was used during group allocation, data collection and/or data analysis

Please outline where this information can be found within the submission (e.g., sections or figure legends), or explain why this information doesn’t apply to your submission:

Sample allocation into drug treatment and other comparison groups is described in “Material and Methods” and figure legends. Samples were not randomized, but occasionally we shuffled the order of applying drug treatments to ensure observed effects were not due to timing. For drug applications, control and treatment samples were paired so that the same cell or field of view was measured in both conditions. All experiments were performed in inbred mice in which comparisons were made between animals of the same strain, differing only by genotype when comparing between wild type and knockout.

**Additional data files (“source data”)**

- We encourage you to upload relevant additional data files, such as numerical data that are represented as a graph in a figure, or as a summary table
- Where provided, these should be in the most useful format, and they can be uploaded as “Source data” files linked to a main figure or table
- Include model definition files including the full list of parameters used
- Include code used for data analysis (e.g., R, MatLab)
- Avoid stating that data files are “available upon request”

Please indicate the figures or tables for which source data files have been provided:

Source data has been generated for Figures 1-6 as well as their figure supplements. This data has been provided in tabular .xlsx format. It has also been uploaded to an accompanying Dryad directory along with MATLAB code which extracts the relevant data and plots figure panels. For statistical analyses, R scripts that reference the source data file for each figure are included in the linked Dryad directory as well.
